# Cancer prevention in people experiencing homelessness: ethical considerations and experiences from the CANCERLESS project

**DOI:** 10.3389/fpubh.2024.1371505

**Published:** 2024-04-08

**Authors:** María del Valle Coronado-Vázquez, Rosa Gómez-Trenado, Beatriz Benito-Sánchez, Jaime Barrio-Cortes, Alejandro Gil-Salmerón, Miguel Amengual-Pliego, Igor Grabovac

**Affiliations:** ^1^Healthcare Center Las Cortes, Gerencia 1 Healthcare Center Las Cortes, Gerencia Asistencial de Atención Primaria, Madrid, Spain; ^2^Facultad de Medicina, Francisco de Vitoria University, Madrid, Spain; ^3^B21-20R Group, Instituto Aragonés de Investigaciones Sanitarias, Universidad de Zaragoza, Zaragoza, Spain; ^4^Foundation for Biosanitary Research and Innovation in Primary Care (FIIBAP), Madrid, Spain; ^5^Health Work Department, Complutense University of Madrid, Madrid, Spain; ^6^Faculty of Health, Camilo José Cela University, Madrid, Spain; ^7^Gregorio Marañón Health Research Institute, Madrid, Spain; ^8^Research Network on Chronicity, Primary Care and Prevention and Health Promotion, Carlos III Health Institute, Madrid, Spain; ^9^International Foundation for Integrated Care, Oxford, United Kingdom; ^10^International University of Valencia, Valencia, Spain; ^11^Complutense University of Madrid, Madrid, Spain; ^12^Department of Social and Preventive Medicine, Medical University of Vienna, Vienna, Austria

**Keywords:** homelessness, vulnerability, cancer prevention, autonomy, equity, public health, ethics, care

## Abstract

The incidence of cancer in Europe has been increasing in recent years. Despite this, cancer prevention has remained a low priority in health policies. Cancer is one of the main causes of mortality among people experiencing homelessness, who continue to have difficulties accessing prevention programs. A strategy that has been tested to favor cancer prevention is the health navigator figure. The objective of *CANCERLESS* project is to implement this model among populations experiencing homelessness in four European countries to foster the prevention and early detection of cancer. In this perspective, a presentation of *CANCERLESS* project is made, and its ethical aspects are discussed according to the ethics of public health, the ethics of care, solidarity, relational autonomy, and the social recognition of the virtue of just generosity. The ethical foundations of *CANCERLESS* project are rooted in social justice and in equity in access to health systems in general and cancer screening programs in particular. The ethics of public health guided by utilitarianism are insufficient in serving the interests of the most disadvantaged groups of the population. Hence, it is necessary to resort to relational bioethics that includes the ethics of care and solidarity and that recognizes the moral identity of socially excluded persons, reaffirming their position of equality in society. Relational autonomy therefore provides a broader conception by including the influence of living conditions in decisions. For this reason, the *CANCERLESS* project opts for a dialogue with those affected to incorporate their preferences and values into decisions about cancer prevention.

## 1 Introduction

According to WHO data, cancer is responsible for 16% of deaths that occur worldwide each year (1).

The incidence of cancer in Europe has been increasing in recent years. A 2018 report that analyzed the incidence of 25 types of cancers in 40 European countries concluded that the number of new cases during that year had been 3.91 million (2). Due to the aging and growth of the population, these are expected to increase in Europe to 4.75 million, with an expected mortality of 32% (3).

Despite this, cancer prevention has remained a low priority for governments and even for WHO (4). Investments have been directed to the development of new treatments, which are much more expensive and not accessible to all, instead of to the promotion of preventive measures with proven benefits (5). We cannot make a point to suggest that an acute disease is less priority, but to make sure we do not forget of the ongoing issues, as well as the effects that infectious disease pandemics will have on the health systems and chronic disease care.

Primary and secondary prevention can reduce the economic and personal costs of cancer, preventing physical and psychological problems as well as those derived from treatments (5).

Homelessness is a very common public health problem in middle- and high-income countries. Just in the European Union, it is estimated that 4.1 million people experience homelessness annually, although its real prevalence is difficult to establish due to the lack of a unified concept of “people experiencing homelessness” and to the different methodologies used to calculate this population, rendering comparability across countries difficult (6). In general, in European countries, a “person experiencing homelessness” is defined as someone who sleeps outdoors or in a shelter (7). European Typology of Homelessness and Housing Exclusion (ETHOS) classifies living situations that constitute homelessness or housing exclusion. ETHOS identifies four main categories of living situation: Rooflessness, Houselessness, Insecure Housing, and Inadequate Housing (8).

The profiles of these populations have changed over time. Currently, young people, women and migrant families are the most affected by homelessness (6).

The physical and mental health of individuals experiencing homelessness are worse than that of the general population (9). A lack of financial resources, substance abuse issues exposure to infectious diseases, increased injuries and reduced access to health services contribute to this statistic (10). Psychiatric morbidity is high, with alcohol and drug use disorders, schizophrenia and depression being the most frequent problems (11). Mortality is also higher, mainly due to infections, ischemic heart disease, substance abuse and injuries (12). It is estimated that, on average, the age at which people experiencing homelessness die is approximately 30 years below that of the rest of the population (13).

Cancer has been described as one of the main causes of mortality in people experiencing homelessness (14), with lung cancer being the most frequent in men associated with high prevalence of tobacco smoking and cervical cancer in women due to associated risk factors including limited knowledge of risk factors, limited access to preventive services and tobacco smoking (15, 16). Among these patients, survival at 10 years is also lower (16), in part due to their usually late diagnosis of cancer owing their difficulty accessing health services.

At times, such access is limited by legal problems or discrimination due to one’s homeless status (17). The incidence of advanced cancer is higher among structurally vulnerable populations due to delays in diagnosis and treatment (18). Their structural vulnerability results from poverty, unstable housing, and discrimination based on race and gender (19). In relation to cancer, socioeconomic disadvantages predispose patients to poor medical care (20).

One of the strategies that has been tested to favor the entry of these communities, which experience social exclusion due to homelessness, to health services is the patient navigator model, by whom information is given to people about healthy lifestyles, diagnostic tests and treatments, facilitating their inclusion in screening programs (21). It is a person-centered community health model that has been shown to be effective in improving health outcomes through improved accessibility to health services (22, 23).

The objective of this perspective article is to discuss the ethical aspects of cancer prevention among people experiencing homelessness based on an analysis of the European *CANCERLESS* project.

## 2 Methodology

### 2.1 The CANCERLESS project

First, we described the European *CANCERLESS* project, its objectives, participants, design, intervention, and its applicability in clinical practice.

*CANCERLESS* stands for “Cancer prevention and early detection among the homeless population in Europe: Coadapting and implementing the health navigator model.”

The *CANCERLESS* project has three objectives: (1) to develop person-centered health services that promote the access of people experiencing homelessness to cancer prevention and screening; (2) to implement the health navigator model among individuals experiencing homelessness in order to reduce the burden of cancer and associated costs; and (3) to use the knowledge gained for the transformation of cancer care and the implementation of the health navigator model in Europe.

This study was carried out through a longitudinal cohort of people experiencing homelessness from Madrid, Athens, Vienna, and Cambridge. Participants of the *CANCERLESS* project are people aged 18 or over users of homelessness services, who fall under one of the ETHOS categories and who provide their consent to participate.

In each country, the project is expected to recruit an average of 300 people aiming to measure the effectiveness of the focal intervention before, after 4–6 weeks and at end of intervention.

The intervention consists of the implementation of the health navigator model to bring primary and secondary cancer prevention services closer to social care points and facilitate access to the health system for screening. The health navigator model is a combination of the patient navigator model and the patient empowerment model. It consists of different phases: (1) sociohealth assessment of people and determination of biopsychosocial risk; (2) health education through recommendations for cancer risks and screening methods; (3) facilitation of adherence to the screening program through the use of relational techniques that create and reinforce trust between people experiencing homelessness and professionals; (4) agree to and accompany to appointments for screening, coordinating these with participants, social service centers, and health centers; (5) accompanying patients throughout the entire process until obtaining results through social support; (6) agreeing with the salient professionals to obtain results and negative news reports; (7) follow-up to guarantee care after screening; and (8) to produce agreements with community organizations for greater flexibility in services and/or to generate facilitation channels adapted to people experiencing homelessness.

In the analysis, quantitative and qualitative methods will be combined amid comparisons of different interventions and countries. Health status will be determined with data related to acute and chronic diseases, time of diagnosis, previous participation in cancer screening campaigns, use of health resources, risk behaviors, alcohol and drug use, diagnosis of depression and anxiety [Depression, Anxiety and Stress Scale (DASS)/Brief Symptom Inventory (BSI), self-perceived health (SF-12 Health Survey) and general health status (Short Form of the Self-Administered Multidimensional Prognostic Index, SELFY-MPI-SF) and Cumulative Illness Rating Scale (CIRS)].

In addition, qualitative data will be collected through a quasiexperimental analysis as follows: (1) Determine the causal relationships between “exposure” and “response” (pre-post) to define the causal relationships obtained from the bidirectional analysis of social barrier-determinant impact; (2) Development of facilitators and/or elimination of barriers associated with social determinants on codified navigation actions; (3) Define the adherence rate that allows us to measure the type of performance and time of the professional with regards to the relational objectives; and (4) Delineate the requisite professional profiles and types of skill difficulty concerning adherence.

A cost-effectiveness analysis will be carried out using Monitoring and Assessment Framework for the European Innovation Partnership on Active and Healthy Aging (MAFEIP), which calculates impact indicators such as the incremental value related to age and estimates the accumulated utility based on the cost of innovation and on the improvement in quality of life related to health.

Sociodemographic characteristics and health outcomes will be evaluated across the global sample and separately in each of the countries. The main effects during follow-up will be measured among the total population using an intention-to-treat analysis.

### 2.2 Literature review

Secondly, in order to discuss the ethical aspects of cancer prevention in people experiencing homelessness, a narrative review of the available studies published in PubMed, Web of Science and Scopus was conducted.

The Medical Subject Headings (MESH) terms included were: Homelessness, Prevention, Cancer, and Ethic.

As eligibility criteria, we defined the inclusion and exclusion criteria based on the population and conceptual framework of the study:

- Population: People experiencing homelessness including those individuals living in supportive housing, transitional housing, unstable housing, inadequate housing, and inappropriate housing.
- Conceptual framework: Access to cancer prevention programs (detection of specific types of cancer, such as breast, cervix, and colon).
- Articles included: Studies conducted in any environment/country/health system. No limitations in terms of sex and gender. Original research and reviews (qualitative, quantitative, and mixed method study designs). Gray literature. Articles published until June 2023 were included.
- Exclusion criteria: Any publication other than original research or review. Not having access to the full text of the publication.

## 3 Discussion

The *CANCERLESS* project has been designed based on the hypothesis that a health navigator can improve the access of people experiencing homelessness to cancer prevention and screening programs, acting as a link between this population and health services and thereby overcoming the barriers that these systems interpose.

The following questions are therefore raised: (1) according to the ethics of public health, the focus should be placed on minority populations who are excluded from cancer prevention and screening programs due to the determinants that they present as indicators of social exclusion; (2) it is necessary to resort to the principle of solidarity when designing public health policies for cancer prevention; and (3) autonomy (liberal) is insufficient in its application among people experiencing homelessness. We must resort to relational autonomy, which has a broader vision of the influence of living conditions on decision-making, as well as social recognition and the virtue of just generosity, which respect citizenship and expand the vision of the obligations of the State to achieve it, thereby preventing the social abandonment suffered by people experiencing homelessness.

### 3.1 Ethics of public health and ethics of care

Prevention measures in public health originated in consequentialism, whereby actions are justified based on their consequences and utility. In terms of cancer prevention campaigns, public policies are directed to the benefit of the majority of the population; minority and excluded groups, those who have difficulties accessing health services, are often discarded from these proceedings.

To counteract excessive consequentialism, the integration of virtues into public health decision-making has been proposed (24). According with the ethics of virtues, health policies would be enriched by introducing the perspectives of different kinds of people.

Among the principles of the ethical practice of public health is the recognition of the excluded members of society, such as individuals experiencing homelessness; this is carried out through information and education concerning these health issues (Information Principle of the Public Health Leadership Society) (25). In addition, we must resort to relational bioethics, specifically, to the concept that solidarity and care are moral practices (26). For Jennings, both solidarity and care imply the recognition of others, reaffirming the moral position of marginalized persons as members of society by recognizing their dignity and providing them health and social services according to their needs (26).

This would be reinforced by the ethics of care (27) within ethical caring, which arises in opposition to the lack of natural caring. Care is associated with people’s emotional relationships, which are distributed in concentric circles. At the most intimate level would be found the primary support networks in which care is established by affection. This is followed by the level of the close stranger or informal support and, last, by that of the remote stranger or help from society. People experiencing homelessness have lost their inner circle of care, while their informal supports are ambivalent and unstable due to their transitory situation.

In these cases, the ethical commitment to care extends not only to the State, but also to institutions and citizens, for the common good and solidarity, reflected in legislation as a guarantee of human rights.

### 3.2 Solidarity in public health policies

Currently, the term solidarity is commonly used to refer to the desire to promote the interests of others, even at personal cost (28). Solidarity understood in this way implies reciprocity, just as there are rights toward others, some obligations are also enforceable. From this perspective, investing in cancer prevention campaigns would lead to involvement in healthy lifestyles or, if not, exclusion from these programs. However, in public health, solidarity action cannot depend exclusively on reciprocity (29) because decisions about health are not isolated from the social context (30). This is the case in the prevention of cancer among people experiencing homelessness, whose choice of healthy lifestyles can be clearly limited by the social determinants they present and their life histories.

In this sense, solidarity implies the recognition of the moral identity of vulnerable individuals, reaffirming their position of equality in society. Solidarity and care implicitly recognize the other as a subject and help society provide resources and services to improve their health (26). In relation to medical care, Carol Goult identifies the structural injustices that still exist, even in solidarity health systems such as those in Europe (31). This recognition connects solidarity with justice (*solidarity of networks*), giving it a practical sense while positioning it as the need to fight to reduce social inequalities in health (31). This solidarity dispenses with taking measures that support those who have limited access to health services.

Their lack of economic resources deprives people of the possibility of achieving the capacities that are considered valuable, such as good health (32), which N. Daniels deems unacceptable and unfair (33). Poverty, homelessness, and discrimination based on race, gender, etc., are not isolated categories. Their intersectionality generates complex social inequalities. This is what happens to individuals experiencing homelessness, among whom poverty, mental health problems, damage related to substance use, racism, violence and cognitive disabilities intersect (34). This situation makes their access to health services even more difficult, which in the case of cancer implies an increase in morbidity and mortality, thus feeding back into their inequities.

Access to health services is key to reducing health inequalities (18). In the case of people experiencing homelessness, their higher mortality from cancer is not only the result of individual behaviors but is also related to their difficulty accessing cancer prevention programs (16). In this way, the responsibility for their disease is not only on these individuals but also on the functioning of the health systems.

In addition to interventions in social conditions, to advance equity, changes are needed in the health system that recognize and promote access to services for people with social vulnerability. This is the goal of the *CANCERLESS* project: supporting individuals experiencing homelessness so that they can have the same cancer prevention and screening opportunities as any other member of society.

However, on many occasions, the lack of public support limits the implementation of reforms aiming to reduce health disparities (35). At the base of this is what has been called the *status quo* bias, a position of aversion to change motivated by the benefits that individuals receive from the system without worrying about the damage they cause (36), justified in a liberal system, which considers social inequalities the product of the choice to lead an unhealthy way of life (37).

### 3.3 Relational autonomy for participation in cancer screening

It has often been suggested that in cancer screening programs, there is institutional pressure to increase the participation of individuals, with autonomy in decisions being underprioritized (38).

Autonomy has a strongly individualistic character, underscoring the decisions of people regardless of their circumstances, that is, their ability to exclusively make a choice without coercion or to make an informed decision. This notion has recently been questioned by bioethics following the impacts of the COVID-19 pandemic (39). Both concepts lack the relational sense that autonomy should have, considering the influence of social determinants on decision-making (40). Relational autonomy, although not yet well conceptualized, implies the recognition of the historical, social and cultural context of people making such a choice (41).

In this project, we suggest that decision-making on whether to participate in cancer screening programs should take into account the conditions in which people experiencing homelessness live. This implies respect for their values via a relational vision of autonomy that aims to involve participants in discussions about what best suits their personal preferences (42).

### 3.4 Social recognition and the virtue of just generosity

We cannot address the problem of social exclusion and foster the early detection of cancer among people experiencing homelessness, without rethinking ethics according to the verification of the fragility and exclusion of this population, specifically, by establishing that we are all interdependent in some stage of our lives.

MacIntyre argues that the virtue of just generosity is essential for knowing how to treat people who require support; it assumes that oneself has been, can be and will be a subject in need of care from others (43). Acting with just generosity requires that one operates via the attentive and affectionate consideration of the other. When this does not happen, it is always an indication of a moral defect, of an inability to act as duty requires.

Therefore, just generosity is not about helping people experiencing homelessness but concerns the recognition of their citizenship, ensuring that the State must be fair in the distribution of tasks to achieve this, thereby preventing the social abandonment suffered by individuals experiencing homelessness.

## 4 Conclusion

The ethical foundations of the *CANCERLESS* project are rooted in social justice and in equity in access to cancer screening programs for individuals experiencing homelessness.

The ethics of public health, originating in utilitarianism, are insufficient for serving the interests of the most disadvantaged groups in any population.

It is necessary to resort to a relational bioethics that includes solidarity and that recognizes the moral identity of socially excluded persons, reaffirming their position of equality in society.

The recognition that structural injustices still exist in health systems links solidarity with justice and positions it alongside the need to fight to reduce health inequalities.

Relational autonomy provides a broader conception of decision-making by considering the living conditions of people experiencing homelessness. Therefore, it is a more appropriate concept with regards to decision-making on participation in cancer screening programs. However, the State must generate possibilities in the distribution of such tasks to prevent their abandonment and to reduce the impact of this disease among the population experiencing homeless.

The recognition of citizenship and the virtue of just generosity can facilitate the equitable treatment of the population experiencing homeless, generating health systems focused on people that address their vulnerabilities.

## Data availability statement

The original contributions presented in the study are included in the article/supplementary material, further inquiries can be directed to the corresponding author.

## Author contributions

MC-V: Conceptualization, Data curation, Formal analysis, Investigation, Methodology, Resources, Supervision, Validation, Visualization, Writing – original draft, Writing – review & editing. RG-T: Conceptualization, Data curation, Formal analysis, Investigation, Methodology, Project administration, Resources, Supervision, Validation, Visualization, Writing – original draft, Writing – review & editing. BB-S: Conceptualization, Data curation, Formal analysis, Investigation, Methodology, Supervision, Validation, Visualization, Writing – original draft, Writing – review & editing. JB-C: Conceptualization, Data curation, Formal analysis, Funding acquisition, Investigation, Methodology, Project administration, Resources, Software, Supervision, Validation, Visualization, Writing – original draft, Writing – review & editing. AG-S: Validation, Visualization, Writing – original draft, Writing – review & editing. MA-P: Supervision, Validation, Visualization, Writing – original draft, Writing – review & editing. IG: Supervision, Validation, Visualization, Writing – original draft, Writing – review & editing.
